# Supplementary material for: Planarian microtubules form a network within muscle and regulate injury-induced genes essential for regeneration patterning
Source: Development. 2025 Oct 16;152(20):dev204669. doi: 10.1242/dev.204669 (PMC12579930; doi:10.1242/dev.204669)
Supplement: Supplementary information [file develop-152-204669-s1.pdf]

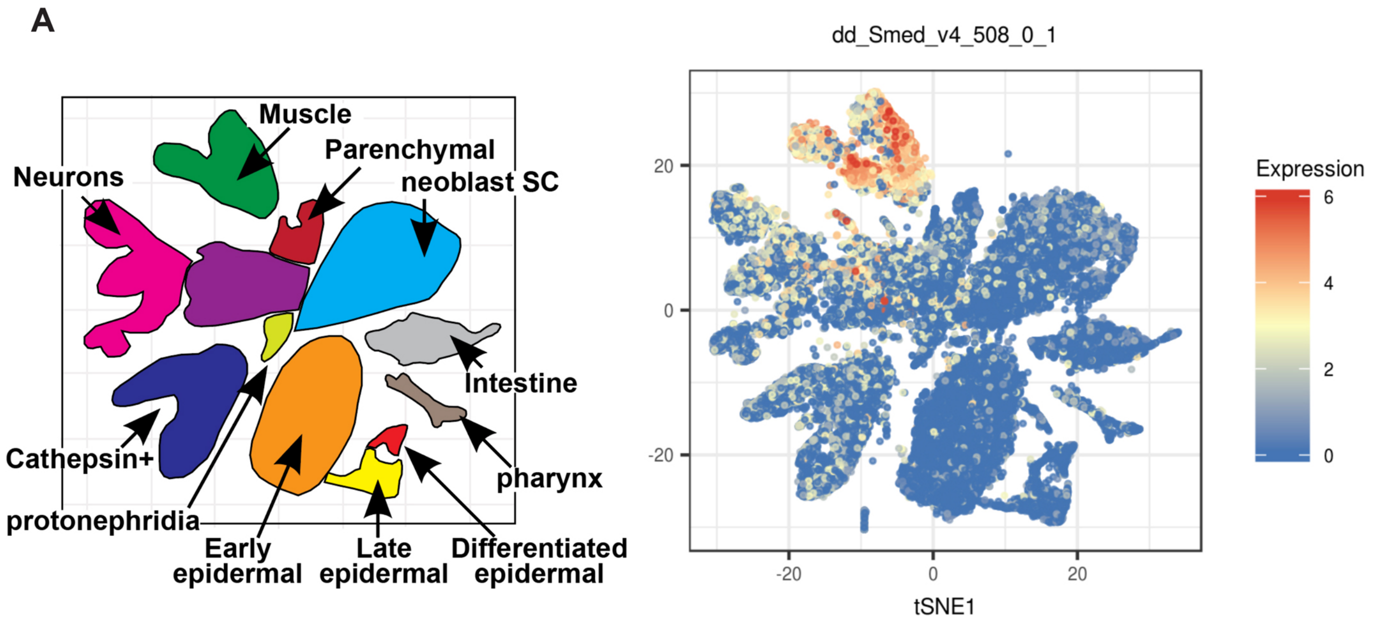

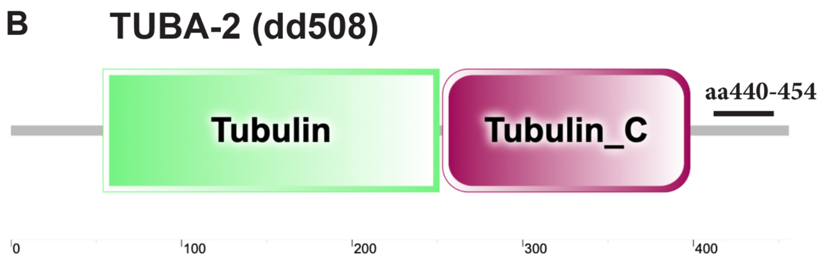

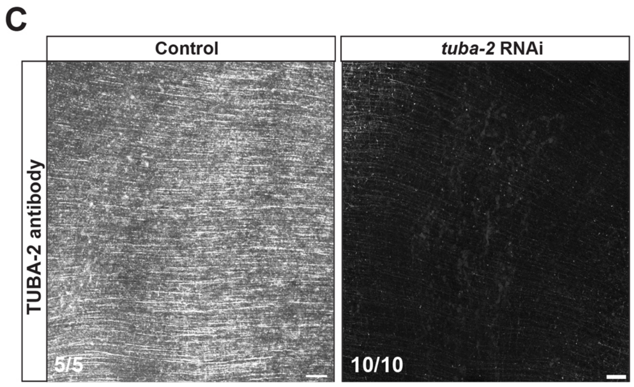

Development • Supplementary information

Fig. S1. TUBA-2 expression and antibody generation

(A) Expression of *tuba-2* detected by single-cell RNAseq in Planarian cell atlas. Left panel shows cell type identification of clusters. (B) Domain structure of TUBA-2 (http://smart.embl- heidelberg.de/) and location of epitope used for antibody generation. (C) Animals were fed control or *tuba-2* dsRNA 6 times over two weeks followed by fixation and immunostaining with the TUBA-2 antibody. Images show maximum projections from pre-pharyngeal regions imaged at identical laser power and gain settings on a Leica Stellaris confocal microscope. *tuba-2* RNAi caused a strong depletion of the muscle expression observed from immunostaining with the TUBA-2 antibody. Scorings indicate the number of control animals which had normal levels of TUBA-2 staining and the number of *tuba-2(RNAi)* animals with strongly reduced or absent TUBA-2 staining. Control RNAi animals had abundant muscle microtubule labeling (5/5 animals), while *tuba-2(RNAi)* animals had either strongly reduced (3/10 animals) or undetectable staining (7/10 animals). Therefore, the anti-TUBA-2 antibody primarily recognizes TUBA-2 protein in fixed tissue.

Development • Supplementary information

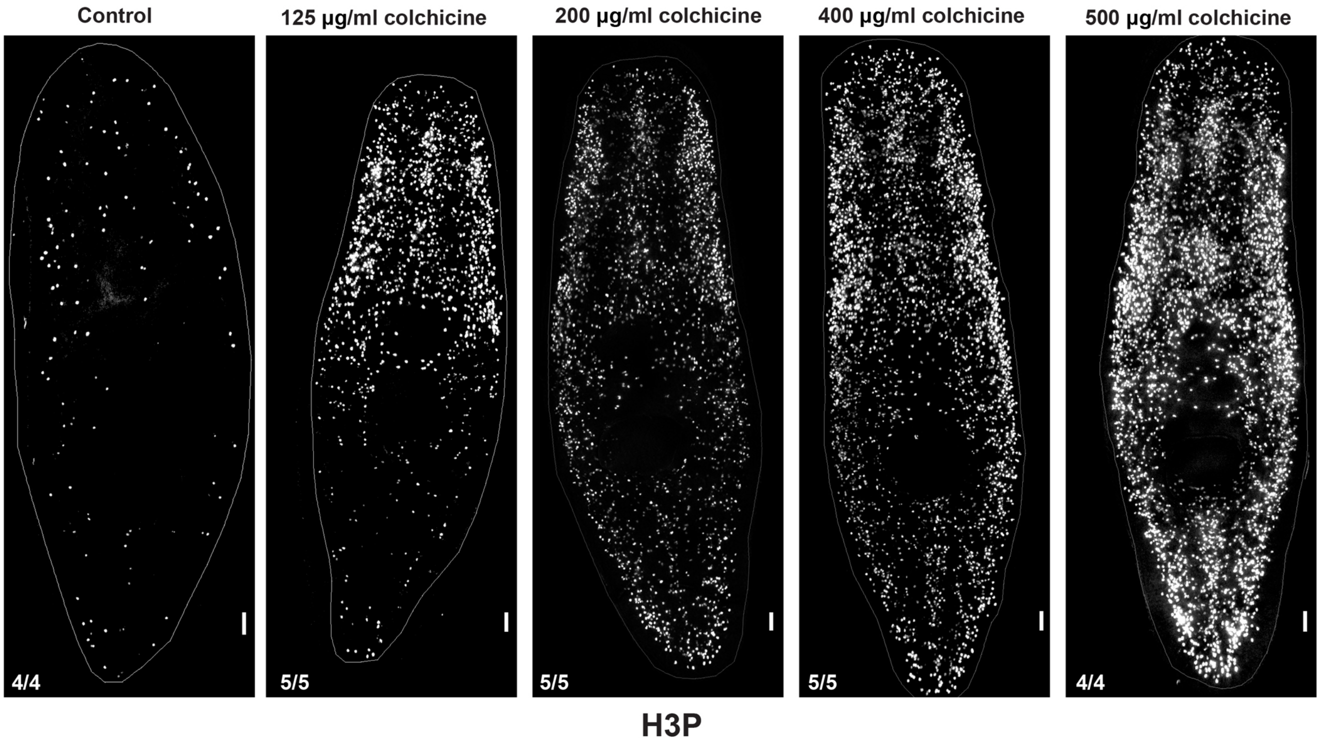

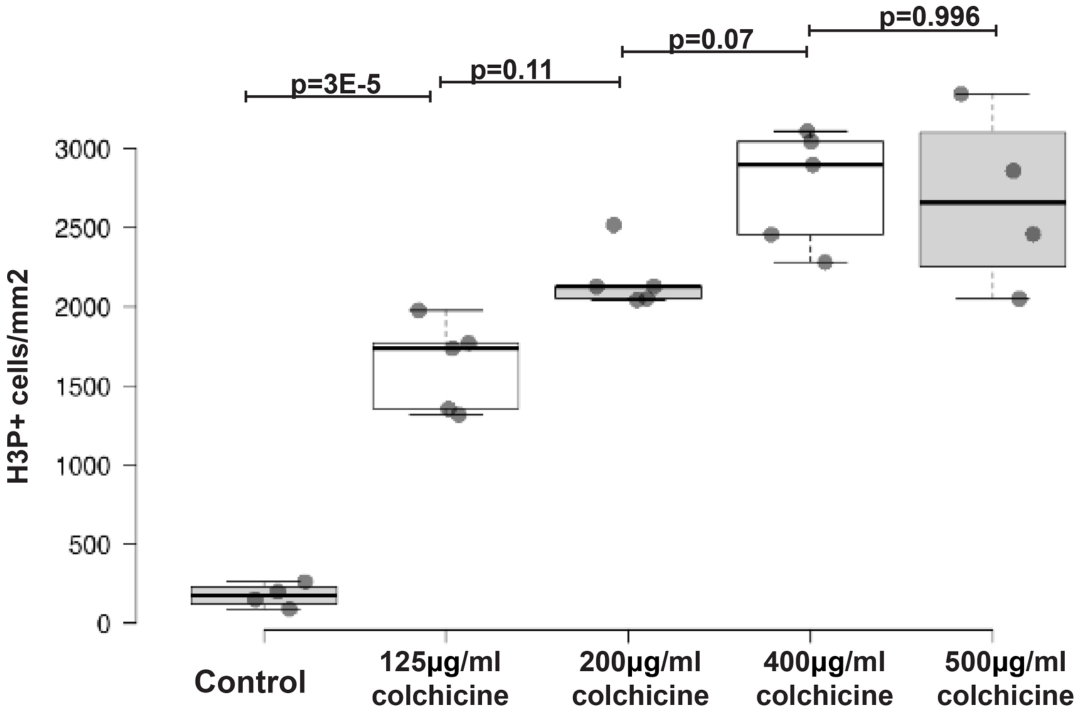

Fig. S2. 125-200 µg/ml colchicine doses cause sub-maximal mitotic arrest.

Intact animals were treated with indicated concentrations of colchicine for 24 hours prior to fixation and immunostaining for H3P (top). H3P+ cells per animal area (mm^2) were quantified (bottom). p-values were determined from 1-way ANOVA and post-hoc Tukey's test. Maximal levels of mitotically arrested cells were observed at 400 and 500 µg/ml colchicine, while 125 µg/ml and 200 µg/ml doses caused sub-maximal increases to H3P+ cells. Scale bars, 50 microns.

Development • Supplementary information

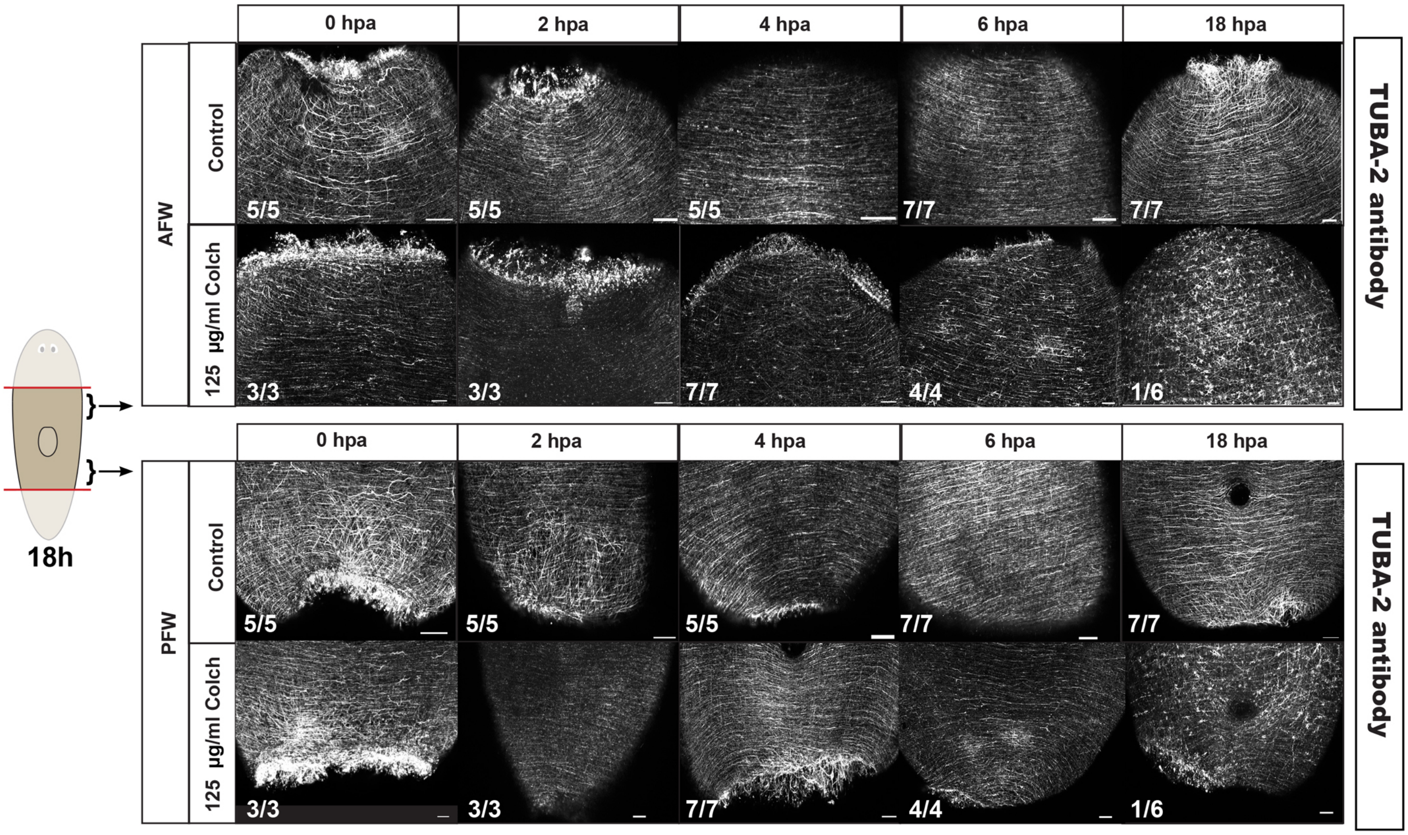

Fig. S3. Regenerating trunk fragments stained with anti-TUBA-2 antibody.

Animals were treated with control media or media containing 125 µg/ml colchicine for 24 hours prior to injury, then amputated and allowed to recover in control media for indicated times followed by Carnoy's fixation and immunostaining to detect TUBA-2 and 6G10 expression. 125 µg/ml colchicine caused loss of fiber expression and accumulation in cell bodies in a pronounced fashion around 18 hours of regeneration. Scale bars, 50 microns.

Development • Supplementary information

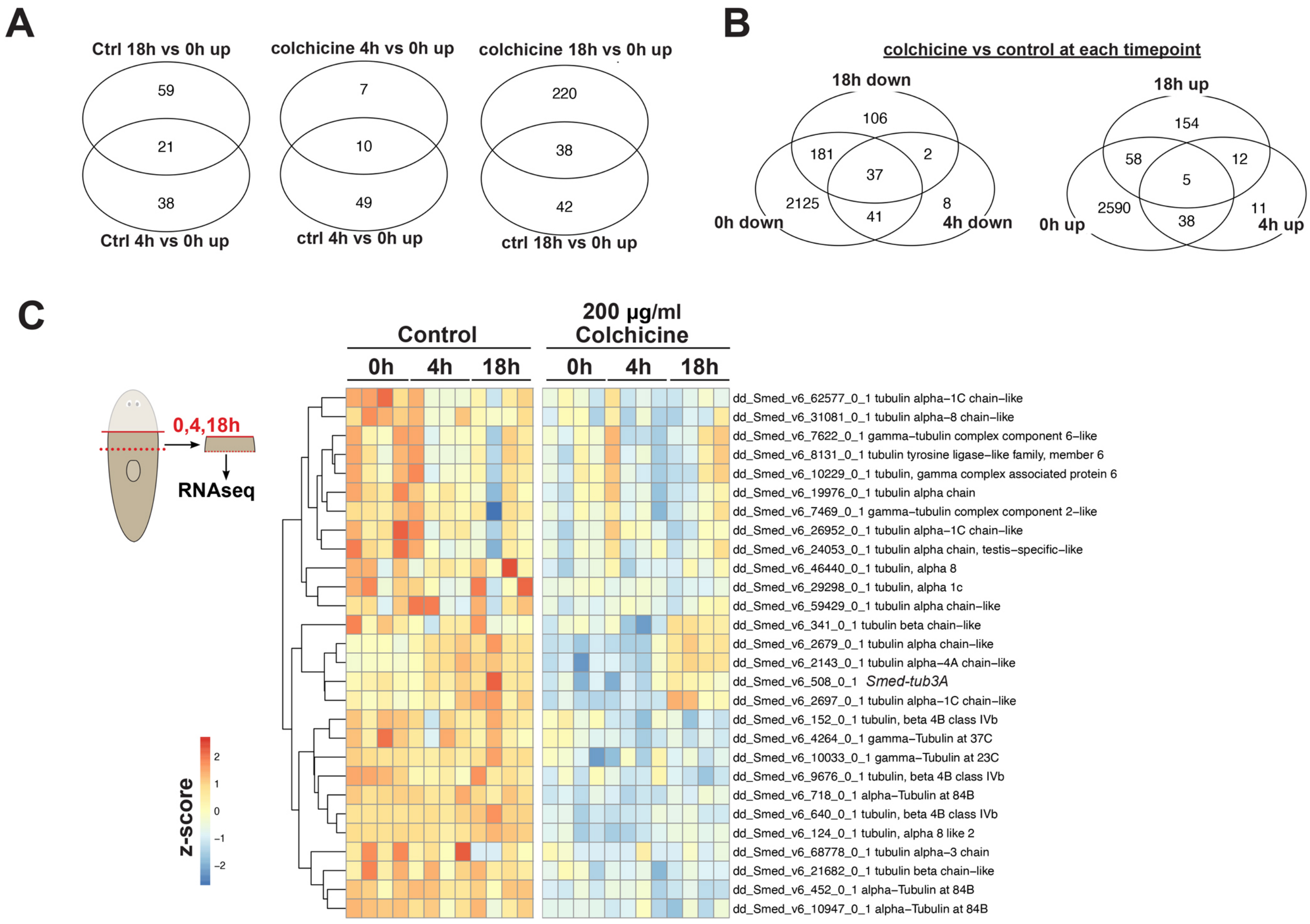

Fig. S4. Additional analysis of RNAseq of colchicine-treated animals.

(A) Summary of number of genes identified as up or downregulated in the RNAseq experiment described in Figure 4 (DEseq padj. < 0.1). Venn diagrams show overlap in the number of genes identified as differentially expressed in control ("Ctrl") or colchicine-treated animals ("colchicine") at the timepoints indicated (0h, 4h, 18h) and whether they were up or downregulated. Left, overlap of number of genes upregulated in control fragments at 4 hours versus 0 hours, compared to control fragments at 18 hours versus 0 hours. Middle, overlap of number of genes upregulated between 0 and 4 hours in control animals versus upregulated between 0 and 4 hours in colchicine-treated animals. Right, overlap of number of genes upregulated between 0 and 18 hours in control animals versus upregulated between 0 and 18 hours in colchicine-treated animals. (B) Venn diagrams showing overlap of number of genes commonly downregulated (left) or upregulated (right) due to colchicine treatment at each of the three timepoints. (C) Tubulins and related genes which showed a significant (p.adj < 0.05) decrease in expression at 0 hours due to colchicine treatment, consistent with planarians possessing a system for tubulin mRNA autoregulation in response to chemical depolymerization of microtubules.

Development • Supplementary information

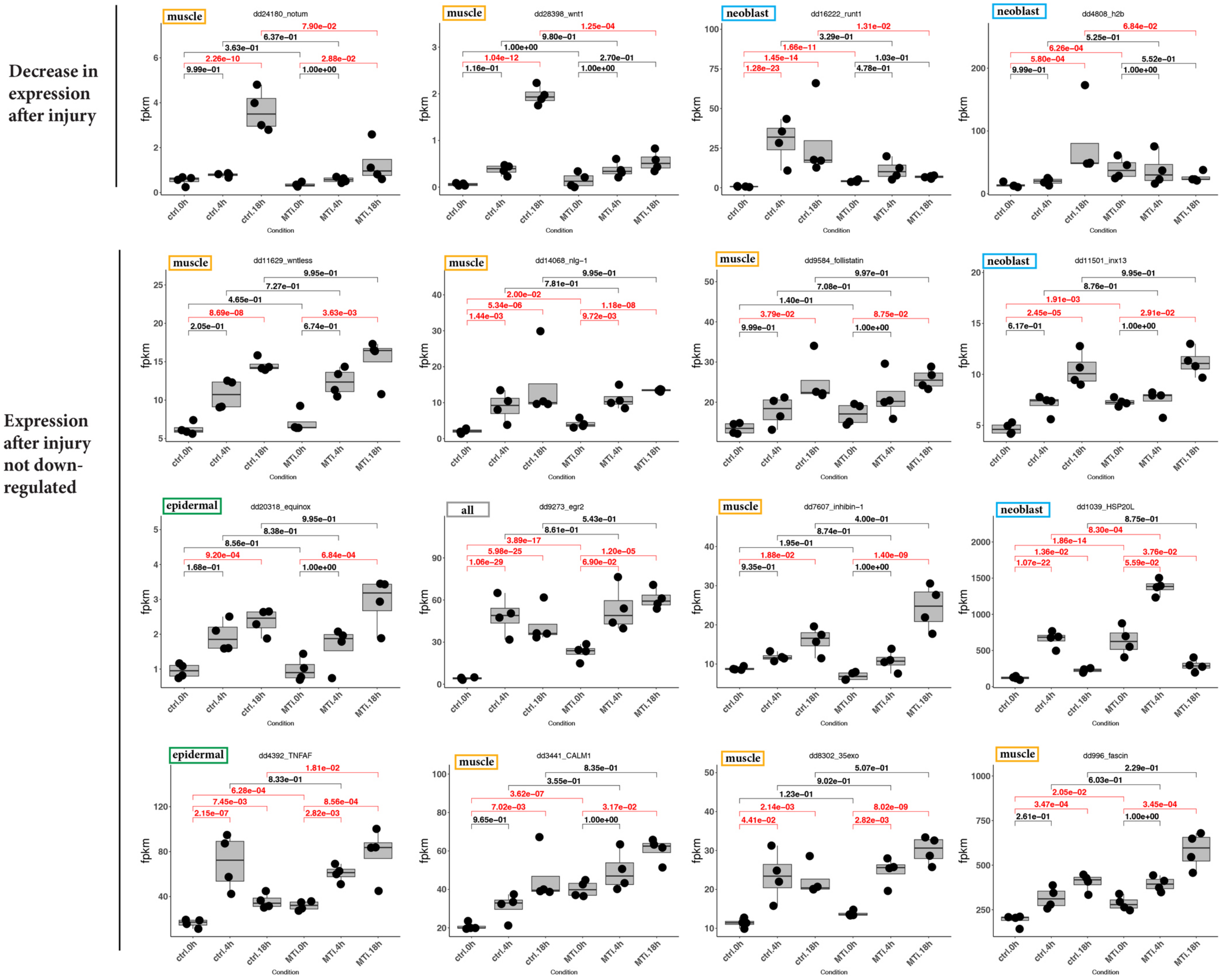

Fig. S5. Expression of wound-induced genes (transcripts per million reads) from RNAseq. Expression levels of wound-induced genes (in transcripts per million reads) from RNAseq. Values above brackets display adjusted p-values for each comparison, and significant differences (padj<0.1, Benjamini-Hochberg correction) are labeled in red, while comparisons with padj>0.1 are labeled in black). Genes were binned into two categories: those whose expression after injury decreased at either 4 hours and/or 18 hours, and those whose injury-induced expression still occurred after colchicine treatment. Colored boxes within each plot describe the known cell type specificity for expression of each gene (Wurtzel, et al. 2015). X-axis labels indicate treatments in control conditions (ctrl.0h, ctrl.4h, ctrl.18h) or colchicine-treated conditions under microtubule inhibition (MTi.0h, MTi.4h, MTi.18h). Dots represent expression levels measured for each biological replicate and boxplots show median and 25%-75% interquartile range.

Development • Supplementary information

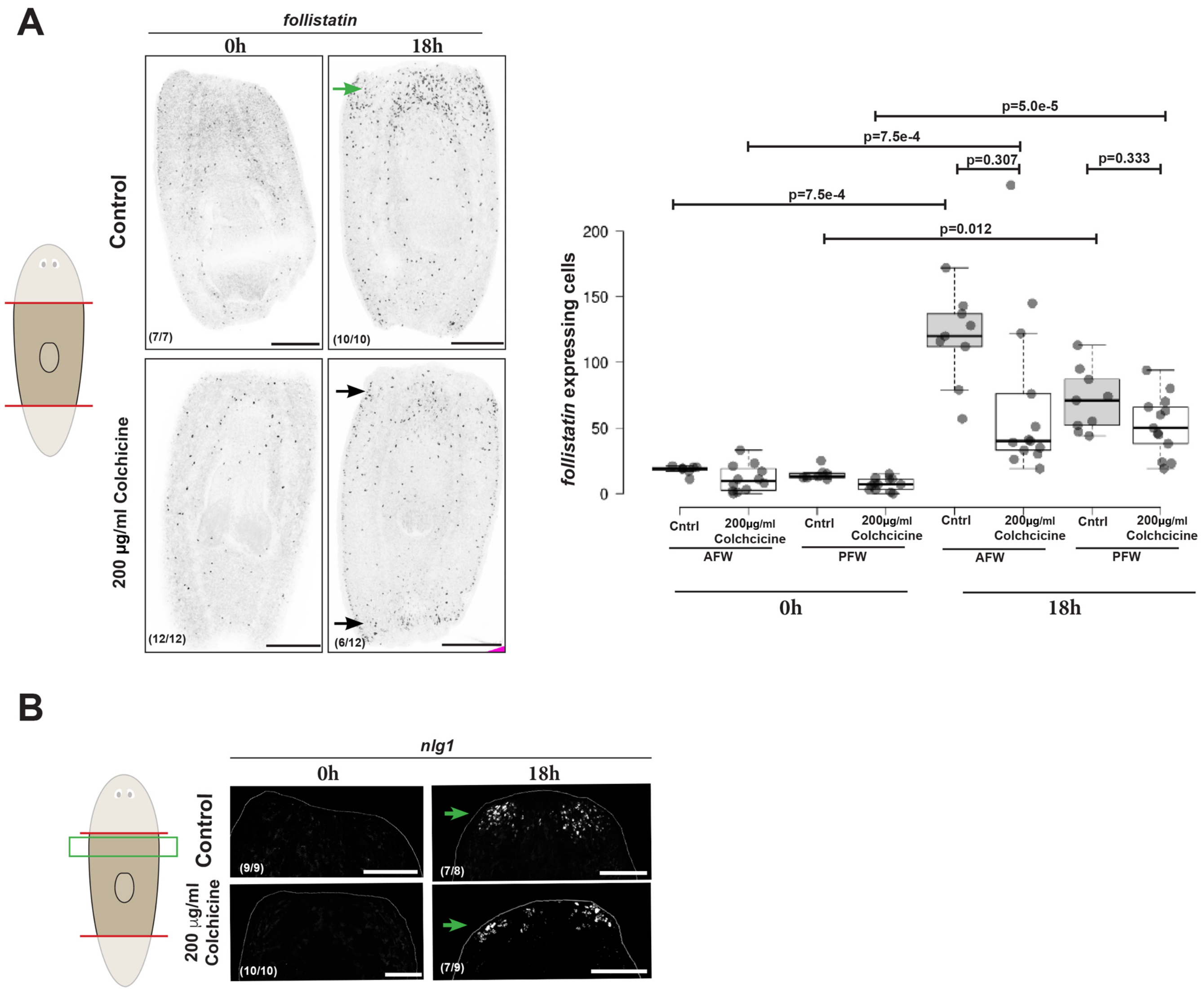

Fig. S6. FISH of injury-induced genes identified by RNAseq as colchicine responsive or nonresponsive

(A-B) Animals were treated with control media or media containing 200 µg/ml colchicine for 24 hours prior to injury, then amputated and allowed to recover in control media for indicated times followed by FISH in animals fixed 18 hours after amputation. (A) Colchicine treatment still enabled expression of *follistatin* though at lower levels at anterior-facing wounds, and levels at posterior-facing wounds were not significantly different from controls (black arrows). p-values were calculated from Kruskal-Wallis test followed by post-hoc Dunn's test to compare multiple samples across conditions. (B) Injury-induced *nlg-1* expression still occurred in colchicine-treated animals. Scale bars depict 200 microns (A), and 100 microns (B).

Development • Supplementary information

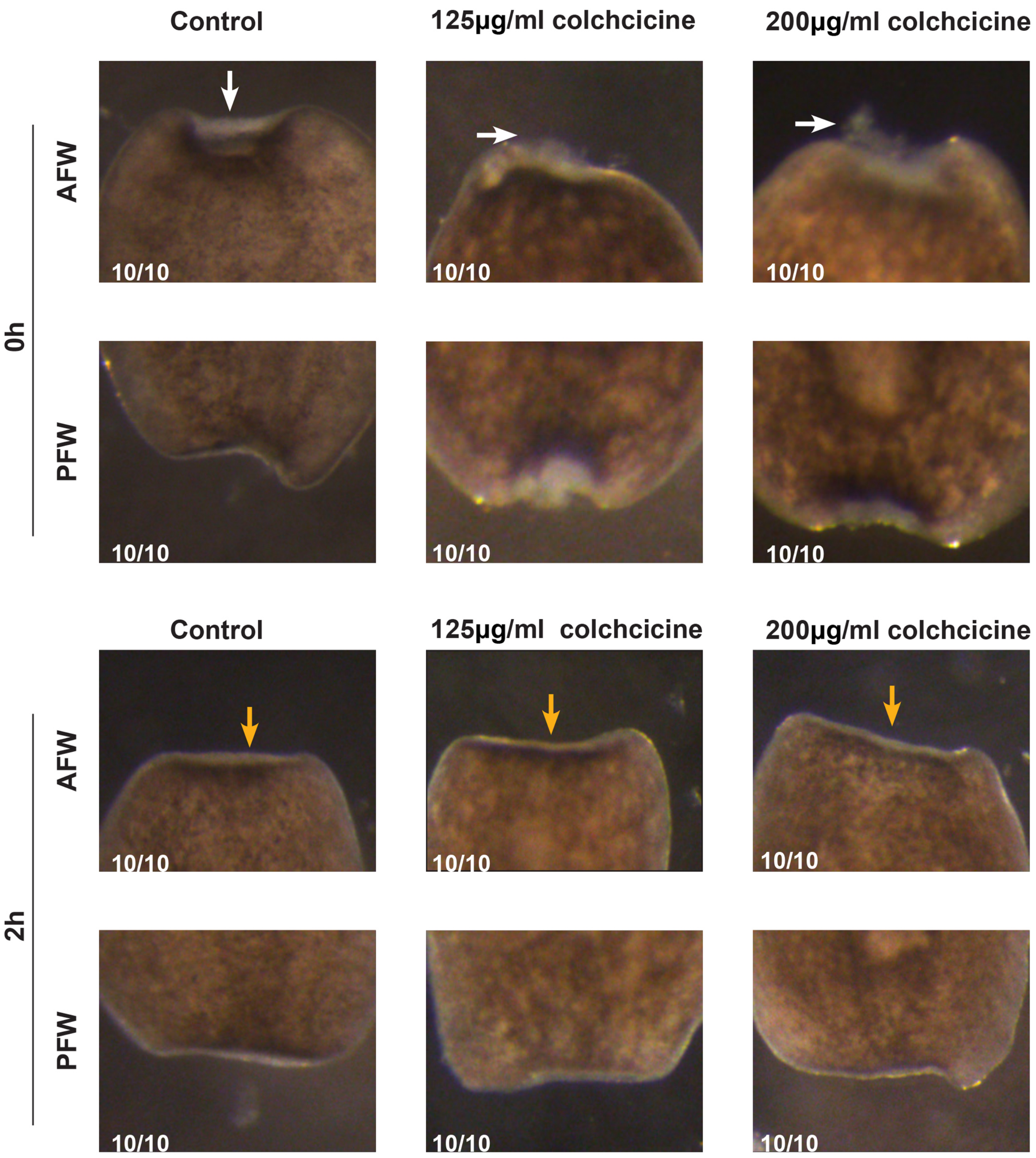

Development • Supplementary information

Fig. S7. Colchicine treatment did not prevent wound healing.

Animals were amputated after 24 hours of treatment with control media, 125 μg/ml colchicine, or 200 μg/ml colchicine, followed by amputation of heads and tails, recovery in control media, and live imaging immediately within the first 5 minutes after surgery ("0h") or at 2 hours post-surgery ("2h"). The anterior-facing wounds (AFW) and posterior-facing wounds (PFW) are indicated. The wound site of freshly amputated animals has a rough appearance (white arrows) with the observation of internal material exiting the wound site. By 2 hours, epidermis seals the wound leading to a smoother appearance and no longer loss of internal material (orange arrows). Colchicine-treated animals healed their wounds within 2 hours, similar to control animals. Scorings indicate the number of animals in the experiment similar to those shown.

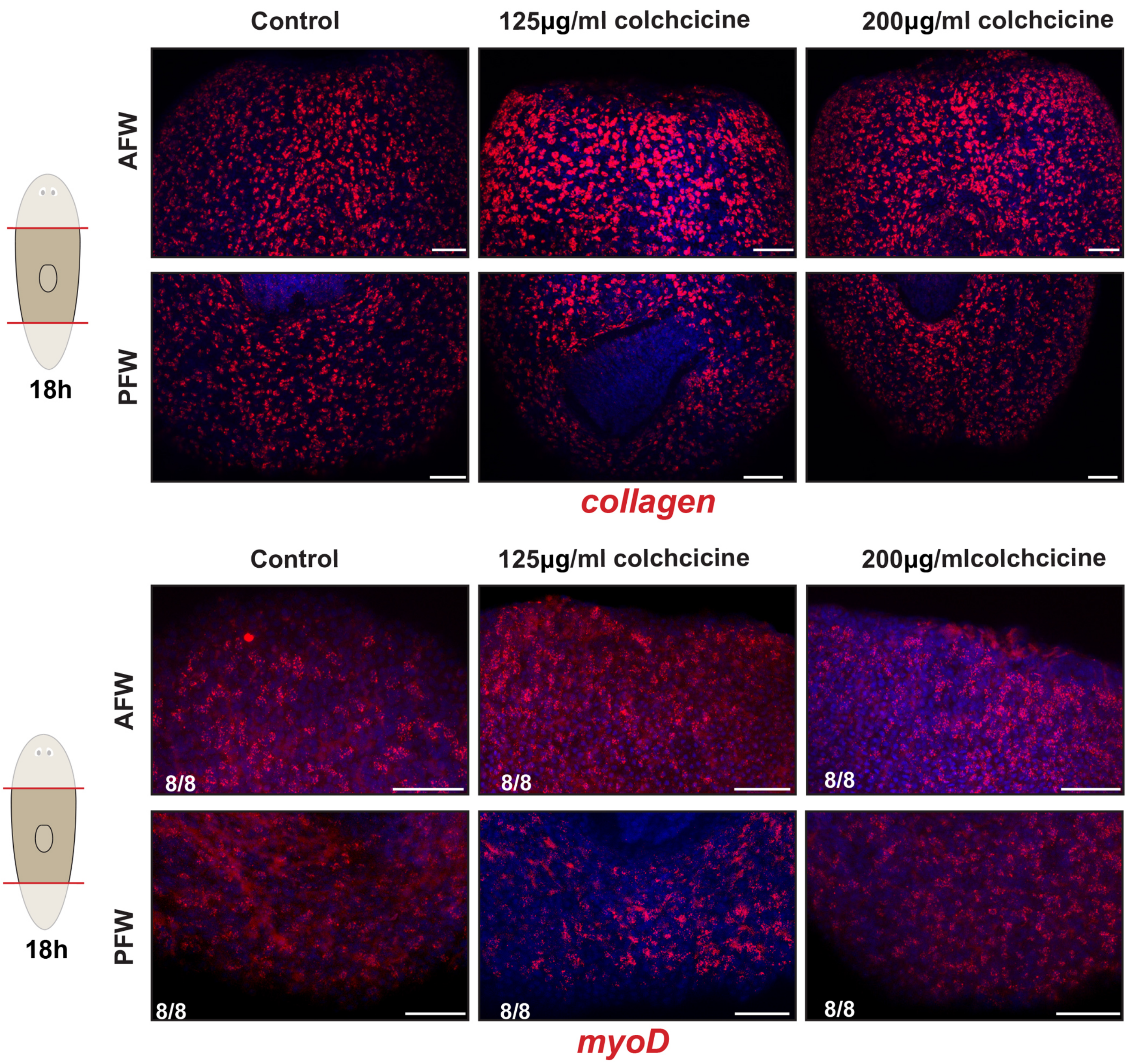

Fig. S8. Colchicine treatment does not eliminate *collagen*+ or *myoD*+ muscle cells.

Animals were amputated after 24 hours of treatment with control media, 125 µg/ml colchicine, or 200 µg/ml colchicine, followed by amputation of heads and tails, recovery in control media for 18 hours, then fixation and staining for muscle cell body markers *collagen* and *myoD.* Colchicine-treated animals still had abundant *collagen* and *myoD*-expressing muscle cells. Scorings indicate the number of animals in the experiment similar to those shown. Scale bars, 50 microns.

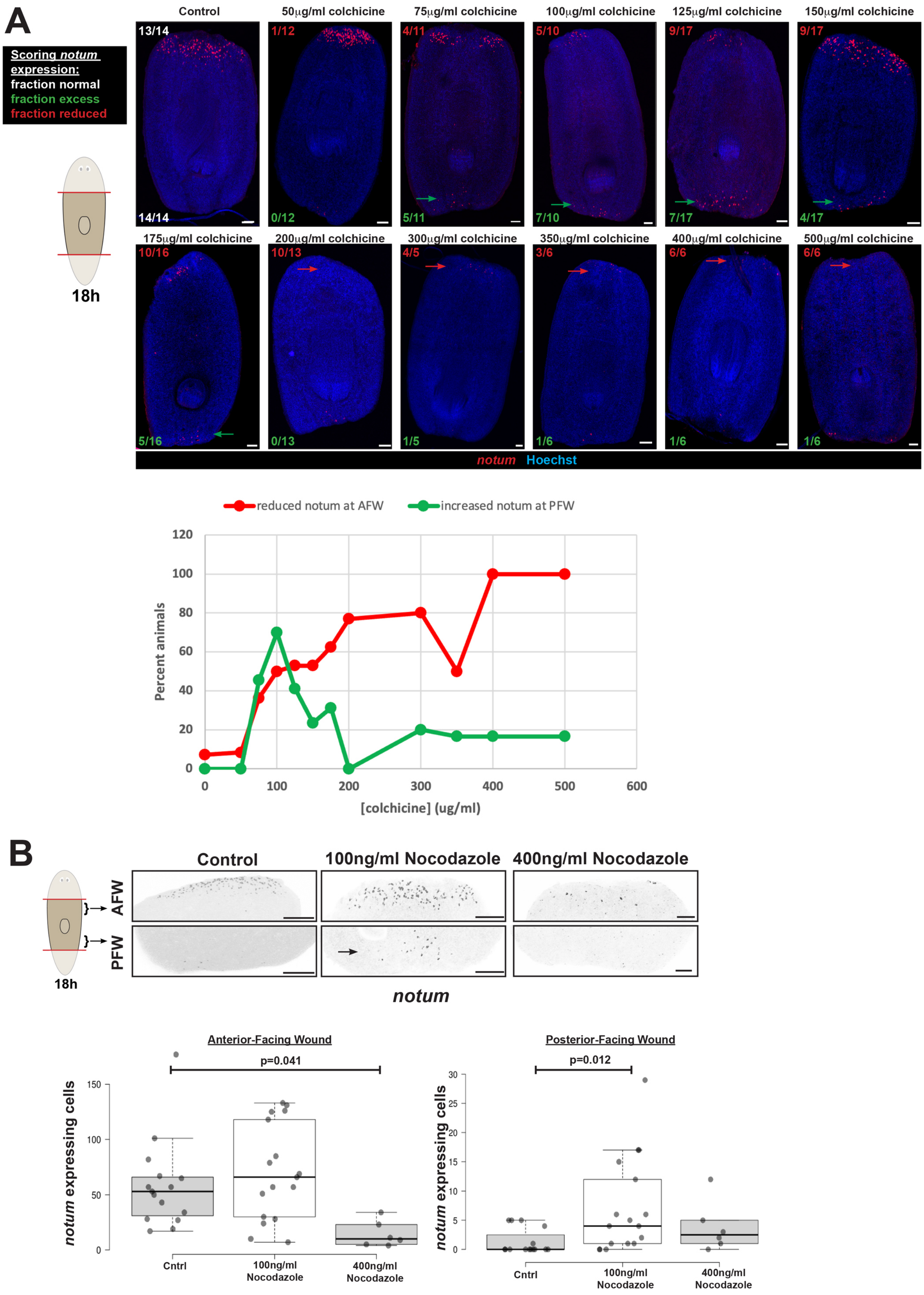

Development • Supplementary information

Fig. S9. Dose series of colchicine effects on *notum* expression and effect of nocodazole treatment on *notum* expression at posterior-facing wounds

(A) Summary of effects from a dose series of colchicine treatment for 24 hours, followed by amputation and recovery in water, then qualitative assessment of *notum* expression behavior at anterior-facing and posterior-facing wounds of regenerating trunk fragments. Scorings of control animals indicate fraction with normal levels of *notum* expression at anterior-facing wounds and normal lack of expression at posterior-facing wounds (white numbers). Scorings of colchicine- treated animals indicate fraction of animals with elevated expression of *notum* at posterior-facing wounds (green numbers) or reduced expression of *notum* at anterior-facing wounds (red numbers). Graph below shows percentages of animals displaying either elevated expression of *notum* at posterior-facing wounds (green) or reduced expression at anterior-facing wounds (red). Maximal penetrance of the phenotype of excess *notum* expression at posterior-facing wounds occurred between 75-125 µg/ml, and 200 µg/ml colchicine was the minimum dose causing at least 70% of animals to have the phenotype of reduced *notum* at anterior-facing wounds. (B) Animals were treated with control media or media containing 100 ng/ml or 400 ng/ml nocodazole for 24 hours prior to amputation and recovery in planarian water, then fixed at 18 hours post-amputation and stained for *notum* expression. 100 ng/ml nocodazole caused an increase to expression of *notum* expression at posterior-facing wounds, while 400 ng/ml nocodazole caused a decrease of *notum* expression at anterior-facing wounds. p-values were calculated from Kruskal-Wallis test followed by post-hoc Dunnett's test to compare multiple samples against a common control condition. Quantifications show numbers of *notum*+ cells counted at anterior- and posterior-facing wounds from individual animals (dots), with overlayed boxplots showing median and 25%-75% interquartile range. Scale bars, 100 microns.

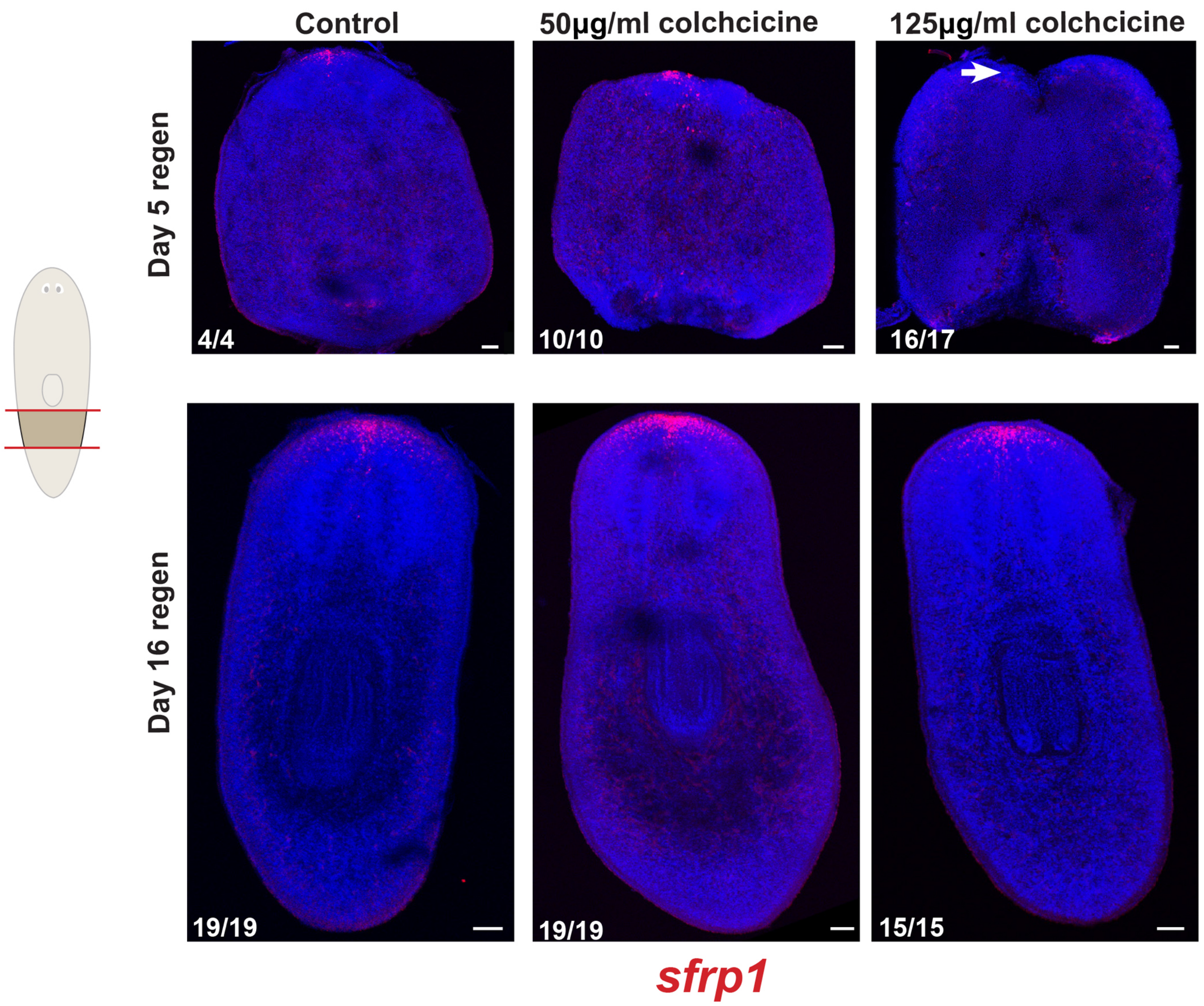

Fig. S10. Colchicine treatment delays anterior regeneration without causing posterior blastemas to regenerate anterior tissue

Animals were treated with control media, 50 μg/ml or 125 μg/ml colchicine for 24 hours prior to amputation of post-pharyngeal transverse fragments, recovery and regeneration in control media, then FISH for *sfrp-1*. Anterior *sfrp-1* was re-established by day 5 of regeneration in control or 50 μg/ml colchicine treatments. 125 μg/ml colchicine treatment caused a delay in head regeneration which prevented *sfrp-1* expression at 5 days (arrow) but enabled expression by 16 days. None of the colchicine treatments resulted in *sfrp-1* expression in the posterior blastema at any timepoint (61/61 animals stained) suggesting blastema head/tail identity was normal in these animals. Scorings indicate the number of animals having a staining pattern resembling the displayed images. Scale bars, 50 microns.

Development • Supplementary information

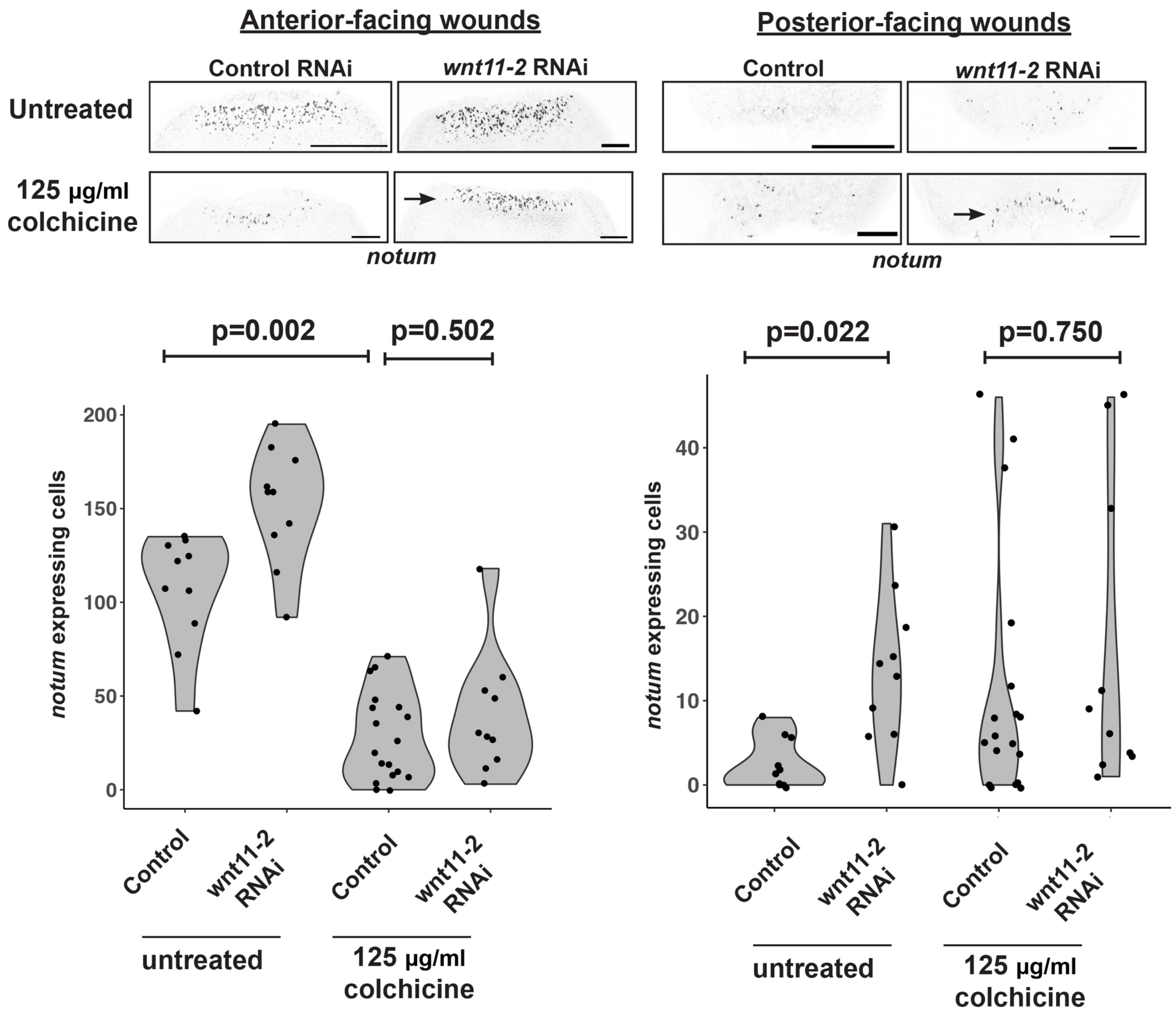

Fig. S11. 125 μg/ml colchicine did not enhance excess *notum* at posterior-facing wounds in *wnt11-2(RNAi)* animals

Animals were treated with *wnt11-2* or control dsRNA for 10 weeks by feeding dsRNA 2-3 times per week, followed by 24 hours of treatment with control media or 125 μg/ml colchicine, amputation of heads and tails, then recovery in control media, followed by fixation and FISH detecting *notum* expression at 18 hours post-amputation. *wnt11-2* RNAi increased *notum* expression at posterior-facing wounds, and we were not able to detect further increases to *notum* expression after simultaneous colchicine treatment (p=0.60). Quantifications show numbers of *notum*+ cells counted at anterior- and posterior-facing wounds from individual animals (dots), with overlayed violin plots. p-values were calculated from Kruskal-Wallis test followed by post- hoc Dunn's test to compare multiple samples across conditions. Scale bars, 100 microns.

Development • Supplementary information

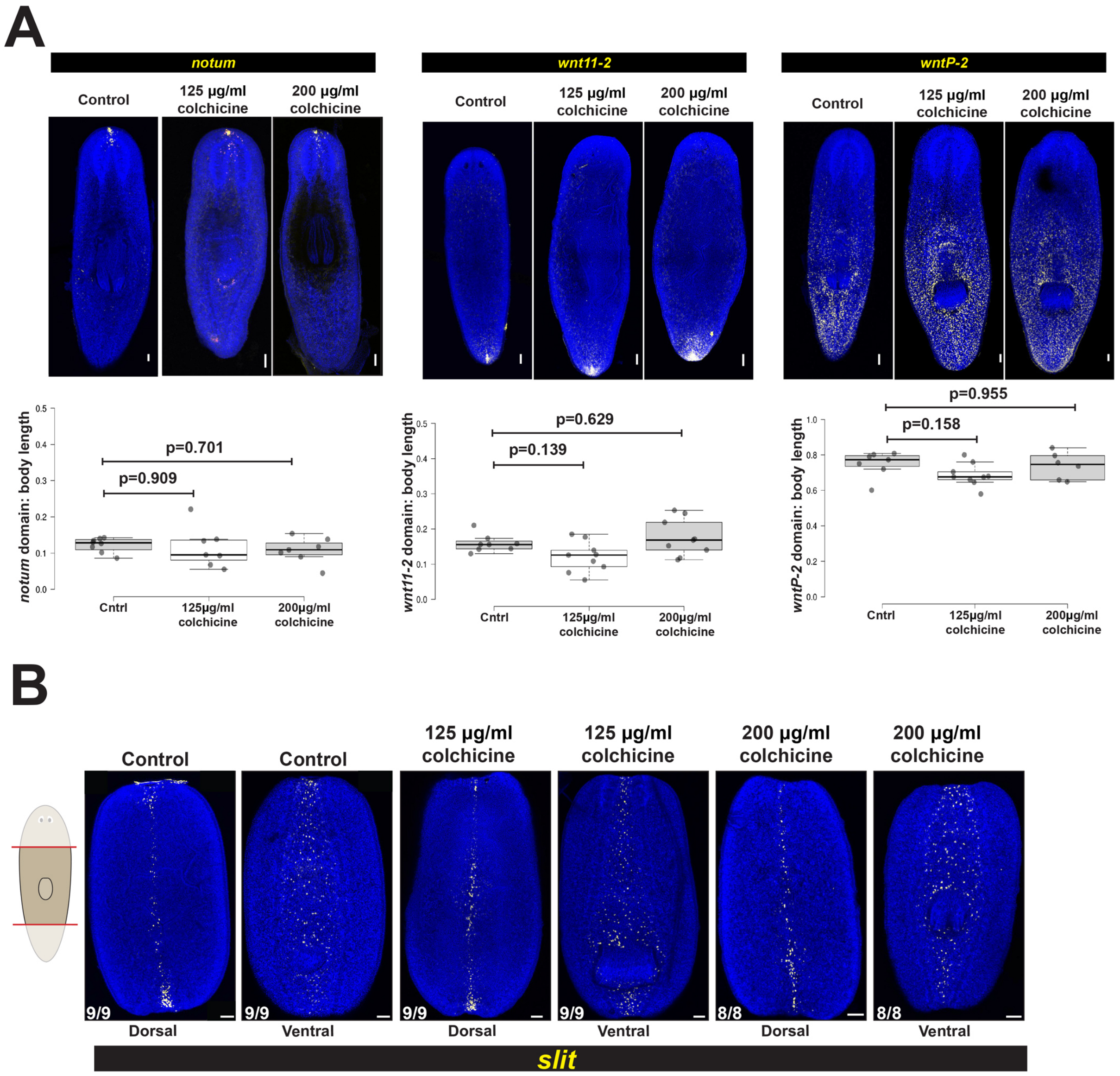

Fig. S12. A-P regionalization of several AP/ML patterning factors was not affected by 125 µg/ml colchicine treatment

(A) Uninjured animals were treated with control media or 125 µg/ml colchicine for 3 days prior to fixation and staining to detect expression of *notum, wnt11-2,* and *wntP-2.* Quantifications show measurements of the relative length of expression for each gene, normalized to total body length. Plots show relative length values obtained from individual animals (dots) with overlayed boxplots. p-values were calculated from Kruskal-Wallis test followed by post-hoc Dunnett's test to compare multiple samples against a common control condition. Colchicine treatment did not cause changes to *notum, wnt11-2,* and *wntP-2* expression domains. (B) Animals were treated with colchicine for 24 hours prior to amputation of heads and tails, recovery in colchicine-free media for 18 hours, then fixed and stained for *slit. slit* expression was not altered by colchicine treatment. Scorings indicate number of animals appearing similar to each image. Scale bars, 50 microns.

Development • Supplementary information

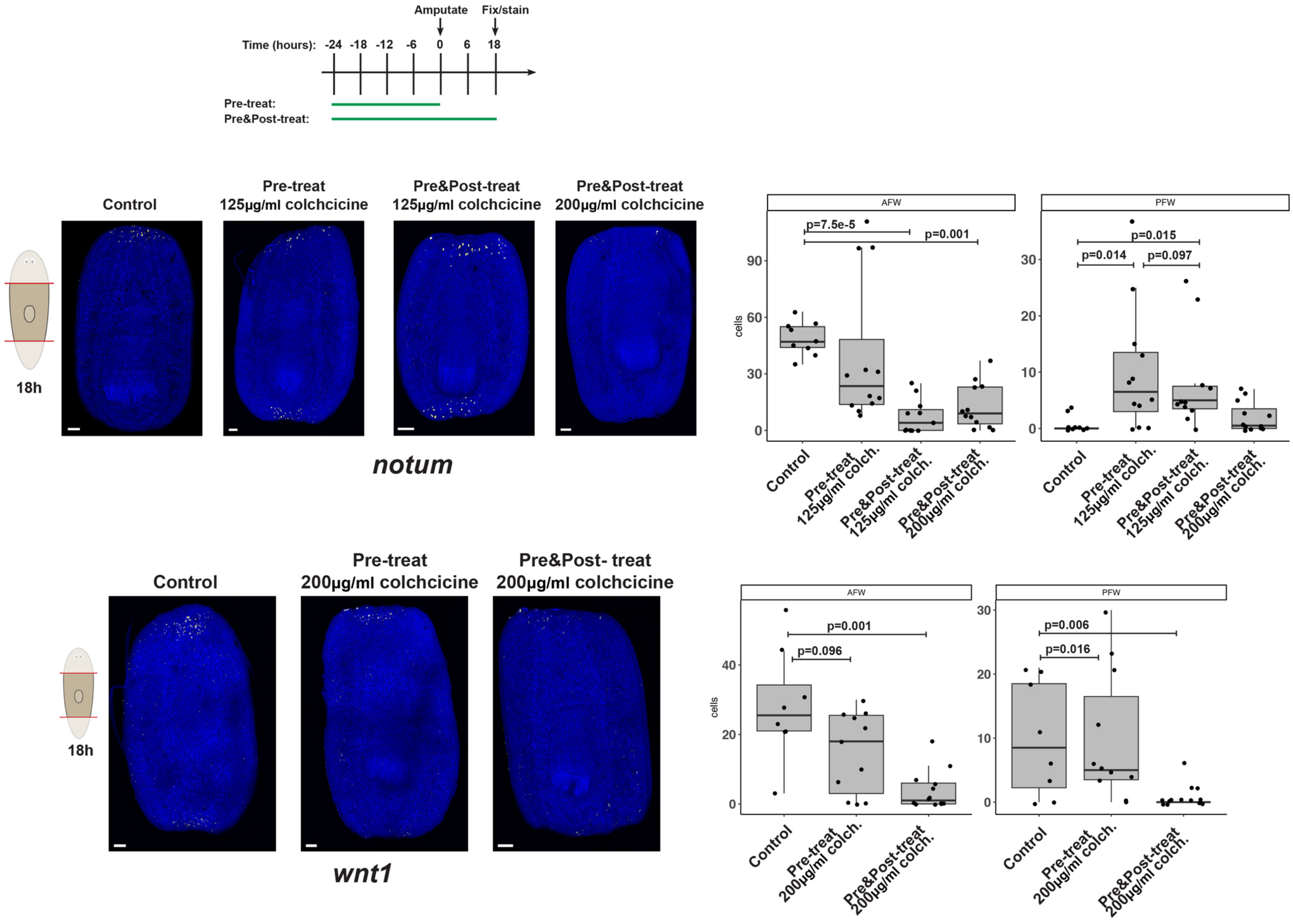

Fig. S13. Testing the influence of recovery in colchicine-free media after microtubule inhibition on *notum* and *wnt1* expression

Animals were subjected to either 125 or 200 µg/ml colchicine, as indicated, for 24 hours prior to injury, followed by either recovery in colchicine-free media ("Pre-treatment") or in colchicine- containing media ("Pre&Post-treatment") prior to fixation and staining at 18 hours then staining for *notum* or *wnt1.* Plots show cell counts at the anterior-facing and posterior-facing wound sites. p-values were calculated from Kruskal-Wallis test followed by post-hoc Dunn's test to compare multiple conditions. Continuous treatment in 125 µg/ml colchicine resulted in elevation of *notum* expression at posterior-facing wounds (p=0.014). Continuous treatment in 200 µg/ml colchicine resulted in lower *notum* expression at anterior-facing wounds (p=7.5e-4). Likewise, continuous treatment in 200 µg/ml colchicine resulted in reduced expression of *wnt1* at both anterior-facing wounds (p=0.001) and posterior-facing wounds (p=0.006). Therefore, the effects of colchicine on expression of *wnt1* and *notum* are not likely to be due to the recovery response following an earlier period of microtubule inhibition.

Development • Supplementary information

Table S1. Annotation of tubulin genes in *Schmidtea mediterranea*

Table enumerates alpha, beta, gamma tubulins in S. mediterranea and associated gene contig names from the Dresden transcriptomes. *tuba-2/dd508* is highlighted.

Available for download at
https://journals.biologists.com/dev/article-lookup/doi/10.1242/dev.204669#supplementary-data

Table S2. RNAseq analysis of wound sites at 0h, 4h, 18h in animals treated with control media versus 200 μg/ml colchicine.

The sub-table tabs display: (1) the data for expression of wound-induced genes "WI genes" across the timeseries, and (2-5) pairwise comparisons of conditions comparing 4 hours control versus 0 hours control (ctrl4h_vs_ctrl0h), comparing 18 hours controls versus 0 hours controls (ctrl18h_vs_ctrl0h), comparing 0 hours colchicine-treated animals and 0 hours controls (COL0h_vs_ctrl0h), comparing 4 hours colchicine-treated animals and 4 hours controls (COL0h_vs_ctrl0h), and comparing 18 hours colchicine-treated animals and 18 hours controls (COL18h_vs_ctrl18h).

Available for download at
https://journals.biologists.com/dev/article-lookup/doi/10.1242/dev.204669#supplementary-data

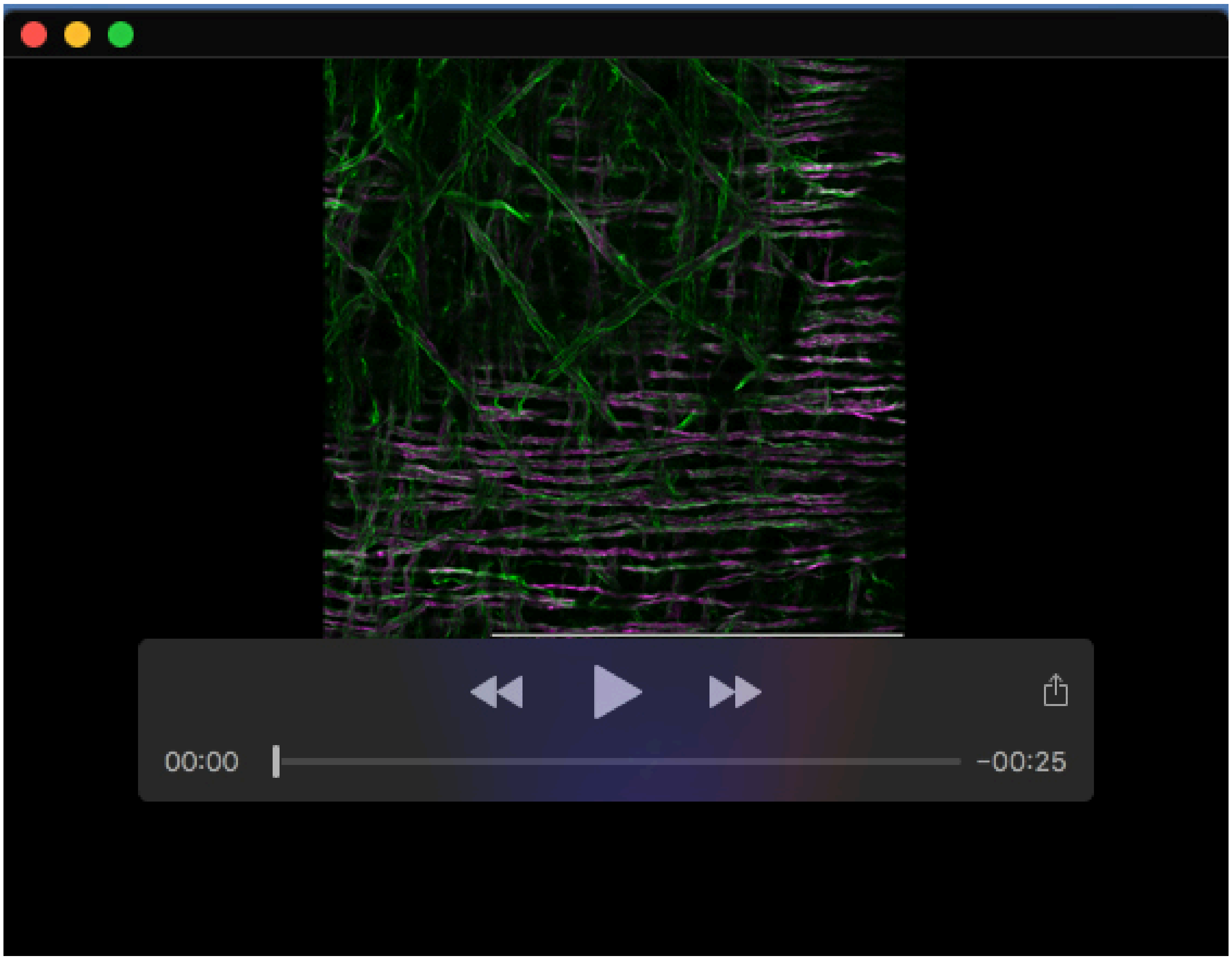

Movie 1. Confocal z-stack using a 20x objective of animals stained with anti-TUBA-2 (green) and 6G10 (magenta) antibodies. Movie progresses from external to internal tissues, showing circular bodywall muscle, then diagonal muscle, then longitudinal muscle. Animal was fixed with Carnoys and primary antibodies detected using fluorophore-conjugated secondary antibodies (TUBA-2 detected with goat anti-rabbit-alexa568 and 6G10 detected with goat anti-mouse- alexa488). Scale bar, 100 microns.

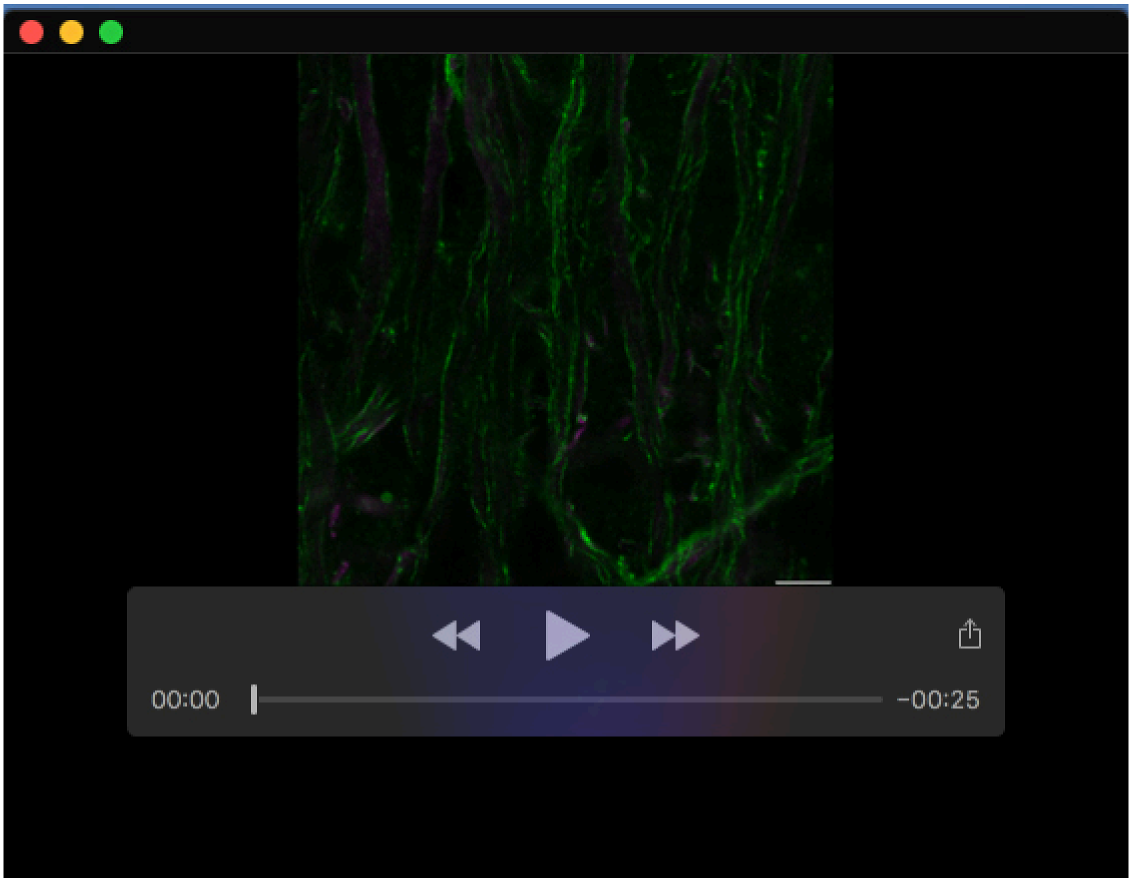

Movie 2. Confocal z-stack using a 40x/1.25NA glycerol objective with 6x confocal zoom of animals stained with anti-TUBA-2 (green) and 6G10 (magenta) antibodies. Movie progresses from external to internal tissues, showing circular bodywall muscle, then diagonal muscle, then longitudinal muscle. Animal was fixed with Carnoys and primary antibodies detected using fluorophore-conjugated secondary antibodies (TUBA-2 detected with goat anti-rabbit-alexa568 and 6G10 detected with goat anti-mouse-alexa488). Scale bar, 10 microns.

Development • Supplementary information

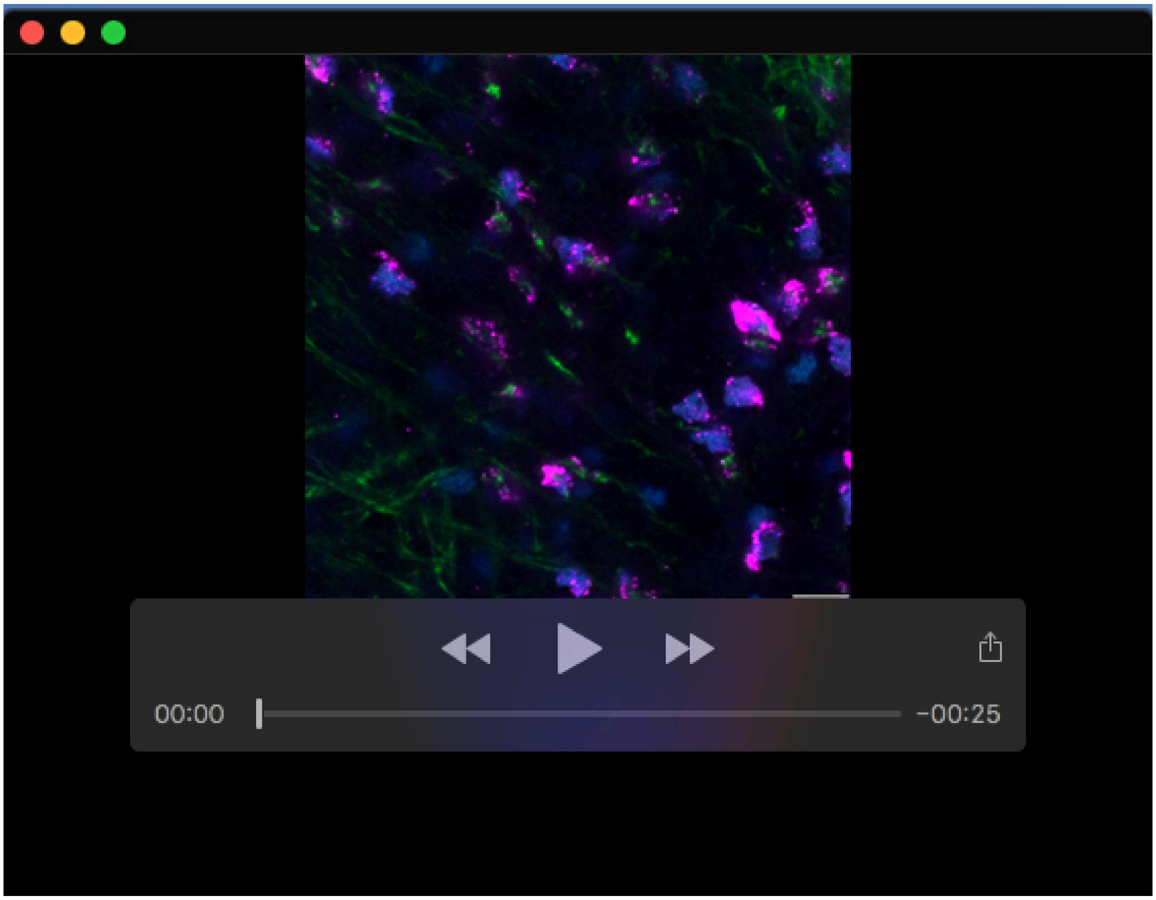

Movie 3. Confocal z-stack using a 40x/1.25NA glycerol objective with 8x confocal zoom of animals immunostained to detect TUBA-2 (green) and FISH to detect *collagen* mRNA (magenta) and nuclei (blue). Movie progresses from external to internal tissues, showing some diagonal muscle, then longitudinal muscle, and image is oriented diagonally with the anterior located at the bottom right. Animal was fixed with NAFA and detection of TUBA-2 and *collagen* mRNA was through sequential tyramide signal amplification of HRP-conjugated antibodies (goat anti-rabbit-HRP for TUBA-2 and anti-digoxigenin-HRP for *collagen*). Scale bar, 10 microns.

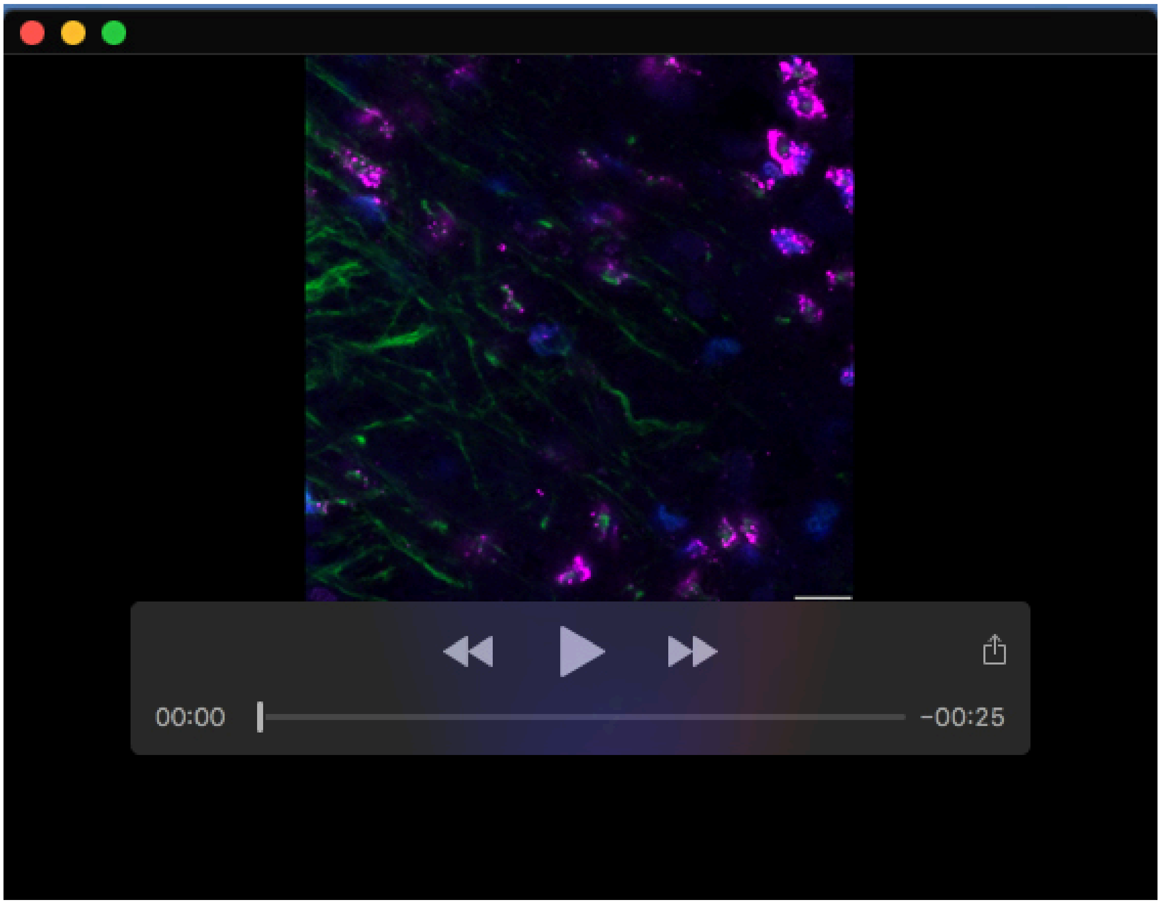

Movie 4. Confocal z-stack using a 40x/1.25NA glycerol objective with 8x confocal zoom of animals immunostained to detect TUBA-2 (green) and FISH to detect *collagen* mRNA (magenta) and nuclei (blue). Movie progresses from internal to external tissues, showing a layer of *collagen*+ cell bodies internally followed by longitudinal muscle, then diagonal muscle, then circular muscle all strongly expressing TUBA-2, followed by nuclei in the epidermis. Animal was fixed with NAFA and detection of TUBA-2 and *collagen* mRNA was through sequential tyramide signal amplification of HRP-conjugated antibodies (goat anti-rabbit-HRP for TUBA-2 and anti- digoxigenin-HRP for *collagen*). Scale bar, 10 microns.

Development • Supplementary information
